# What Autocorrelation Tells Us about Motor Variability: Insights from Dart Throwing

**DOI:** 10.1371/journal.pone.0064332

**Published:** 2013-05-17

**Authors:** Robert J. van Beers, Yor van der Meer, Richard M. Veerman

**Affiliations:** MOVE Research Institute Amsterdam, Faculty of Human Movement Sciences, VU University Amsterdam, Amsterdam, The Netherlands; McMaster University, Canada

## Abstract

In sports such as golf and darts it is important that one can produce ballistic movements of an object towards a goal location with as little variability as possible. A factor that influences this variability is the extent to which motor planning is updated from movement to movement based on observed errors. Previous work has shown that for reaching movements, our motor system uses the learning rate (the proportion of an error that is corrected for in the planning of the next movement) that is optimal for minimizing the endpoint variability. Here we examined whether the learning rate is hard-wired and therefore automatically optimal, or whether it is optimized through experience. We compared the performance of experienced dart players and beginners in a dart task. A hallmark of the optimal learning rate is that the lag-1 autocorrelation of movement endpoints is zero. We found that the lag-1 autocorrelation of experienced dart players was near zero, implying a near-optimal learning rate, whereas it was negative for beginners, suggesting a larger than optimal learning rate. We conclude that learning rates for trial-by-trial motor learning are optimized through experience. This study also highlights the usefulness of the lag-1 autocorrelation as an index of performance in studying motor-skill learning.

## Introduction

In precision sports such as golf, bowling, archery and darts it is crucial to produce accurate ballistic movements of an object towards a goal location. The smaller the variability of the endpoint of the object over repeated movements, the better the performance can be. We will refer to this variability as *performance variability*, which is the inverse of performance consistency. The performance variability depends on various factors. First, noise in the sensorimotor system [1]–[7] places a lower bound on the performance variability. Second, performance variability depends on the kinematics of the limb movement before releasing the object. The kinematics determine how noise in motor signals translates into variability of the object's end position. Skilled sportsmen may produce limb movements with different trajectories than less-skilled sportsmen so as to reduce the variability in the endpoints [2], [8]–[12] and/or the variability in the limb trajectories themselves [13]–[15]. Third, the performance variability depends on the coordination of release parameters such as release speed, release angle and relevant joint angles. Since different limb movements can lead to the same end position of the object, release parameters can co-vary without affecting the object's end position. Numerous studies have shown that humans display such “compensatory variability” in laboratory tasks [11], [16]–[21], and also in sports [10], [22]–[28], where skilled performers are thought to explore and exploit the high dimensionality of the motor system better than less-skilled performers [26], [29].

**Figure 1:**
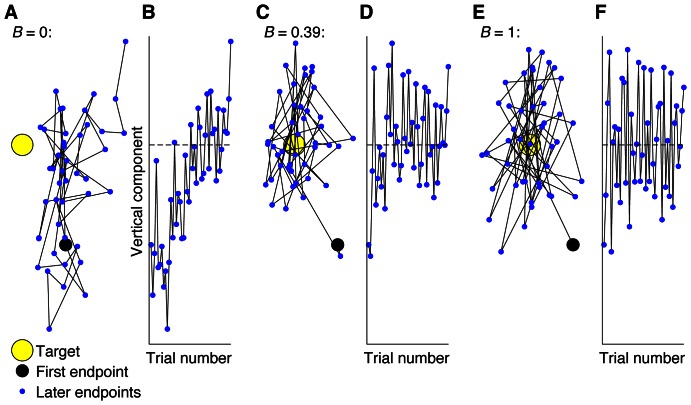
Effect of learning rate on variance and serial correlation. **A** Simulated set of 45 movement endpoints if learning rate *B* = 0 (i.e., no corrections). Consecutive endpoints are connected by lines. Endpoints were generated using the model of van Beers (2009) with *w* = 0.2, where *w* is the proportion of the total effect of motor noise that arises during motor planning. **B** The vertical component of the endpoints shown in **A** plotted as a function of the trial number. **C** The same as in **A**, but now for *B* = 0.39, which is the optimal learning rate for this value of *w*. **D** The vertical component of the endpoints shown in **C** plotted as a function of the trial number. **E** The same as in **A**, but now for *B* = 1 (i.e., correct for the full error). The same set of random numbers was used in **A**, **C** and **E**, only the value of *B* varied. **F** The vertical component of the endpoints shown in **E** plotted as a function of the trial number.

A factor that also affects the performance variability but that has received little attention is the extent to which motor planning is updated from movement to movement based on performance errors. It has been known for many years that feedback from a previous movement is used to update planning of the next movement such that this movement is expected to be more accurate [30], [31]. Studies on repeated reaching movements of the unseen hand to a visual target found that motor planning is corrected by a fixed proportion of the observed error in the previous movement [32], [33]; we will refer to this proportion as the *learning rate* and denote it by *B*. The performance variability depends on the learning rate. If no planning corrections are made (i.e., *B* = 0), an initial error, if present, will persist, which is obviously not a good strategy. There is however another disadvantage. The random effects of noise in the central planning of a motor command [5] accumulate over movements [34]. In the absence of trial-by-trial planning corrections, the endpoint will therefore drift randomly, like a random walk (see Figure 1A), giving rise to unnecessarily large performance variability. An alternatively strategy could be to correct for the full observed error (i.e., *B* = 1). Although the mean endpoint will in that case be on target (see Figure 1E), this is not a good strategy either, because part of the error is not due to incorrect planning but to noise in the execution of the movement (i.e., noise in the relay of the motor command by motoneurons and in the conversion into mechanical forces in muscles [3], [6]. When this strategy is followed, corrections are too large as they correct not only for incorrect planning but also for random effects of noise in movement execution, making it likely that one will end up at the opposite side of the target than in the previous movement (see Figure 1E). This also leads to unnecessarily large performance variability. The performance variability is smallest for intermediate learning rates. The minimum is obtained when the learning rate is large enough to counteract random walks, and small enough to avoid jumping over the target (see Figure 1C).

Not only the variance but also the serial dependence of the endpoints depends on the learning rate. If no corrections are made (Figure 1A), each endpoint is close to the previous one. This can also be seen in Figure 1B, which shows the vertical component of the endpoints in Figure 1A as a function of the movement number. The lag-1 autocorrelation (ACF(1)), which quantifies the correlation between endpoints of consecutive movements, will therefore be positive. If one corrects for the full error (Figure 1E, F), endpoints of consecutive movements tend to be on opposite sides of the mean endpoint, giving rise to a negative ACF(1). For learning rates that lead to a small variance, the ACF(1) will be close to zero, so that each endpoint is statistically independent of the previous one (Figure 1C, D). Van Beers [32] developed a model that made the relationship between learning rate, variance and autocorrelation explicit. We will show here that according to that model, the ACF(1) is always zero for the learning rate that minimizes the variance, independent of the proportions of noise that arise in motor planning and in motor execution.

For the reach task, all participants had ACF(1)s close to zero [32], suggesting that their learning rates were near optimal. This raises the question how they had achieved this. Is our motor system programmed such that it automatically uses the optimal learning rate, or does it learn the appropriate learning rate through experience? To answer this question, we studied dart throwing, as most people have little or no experience with this task, whereas others are highly experienced. We compared expert dart players with beginners in their ability to make repeated throws towards the bullseye. We analyzed the ACF(1) of the positions where the dart landed on the dartboard to determine whether they used the optimal learning rate. If the learning rate is hard-wired, the ACF(1) will be the same for both groups. In contrast, if the optimal learning rate is learned through experience, the ACF(1) can be expected to be near-zero for the experts, and non-zero for the beginners. This hypothesis does not predict the sign of ACF(1) for the beginners. It could be positive for all beginners, it could be negative, but it could also be highly variable across the population, with both positive and negative values in the population. To make sure that potential differences between the autocorrelations of the two groups are the result of their differential dart experience, and not of other differences between the groups, we compared both groups also on a reach task.

## Materials and Methods

### Theoretical relation between ACF(1) and the optimal learning rate

In [32], equations were derived for the endpoint variance Var(**x**) and the lag-1 autocorrelation ACF(1) according to the model developed there:
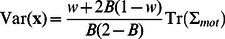
(1)

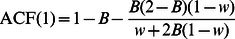
(2)where Σ*_mot_* is a covariance matrix that represents the endpoint variability resulting from noise in the motor system (the combined effect of noise in central movement planning and in peripheral movement execution), Tr denotes the matrix trace, *B* is the learning rate and *w* is the proportion of Σ*_mot_* that arises in movement planning (and 1 – *w* is the proportion that arises in movement execution).

**Figure 2:**
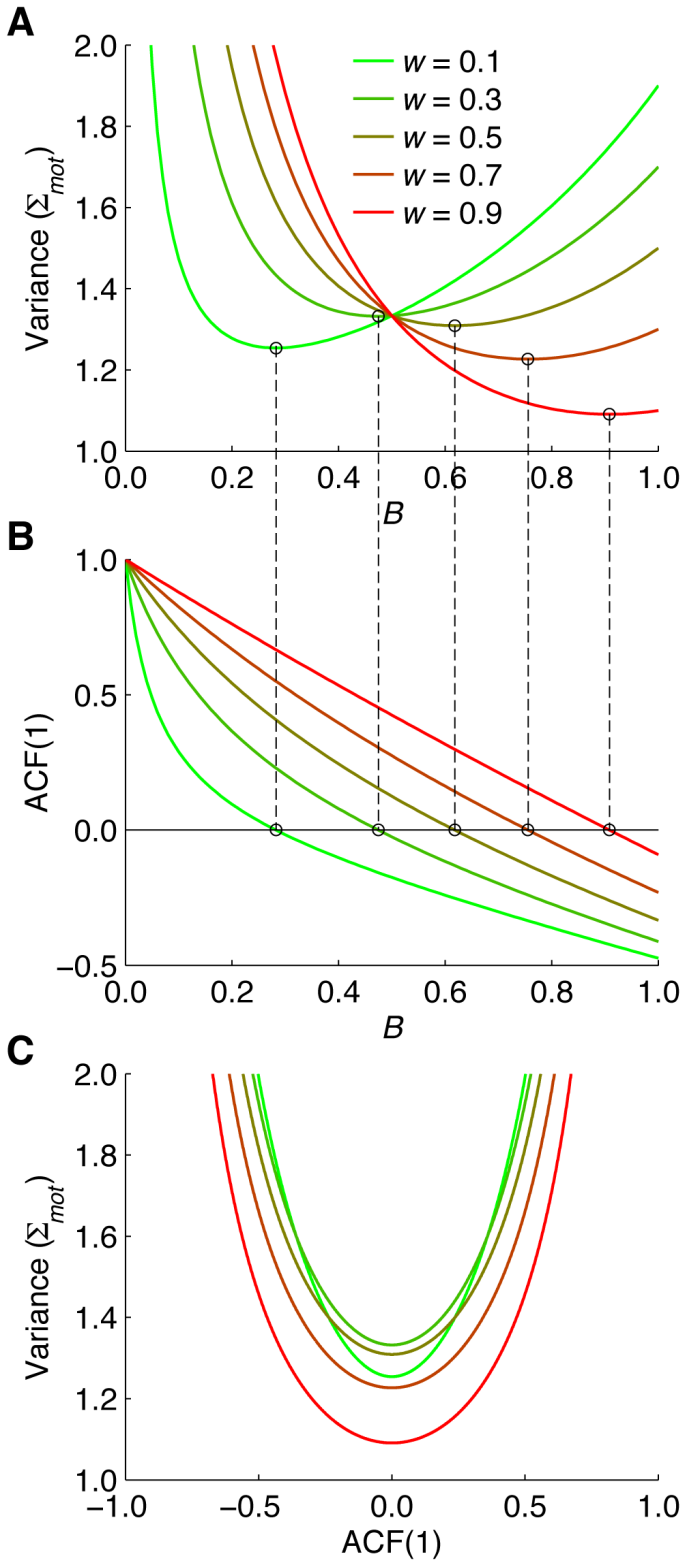
Effect of *w* on the optimal learning rate and the ACF(1). **A** Variance as a function of learning rate *B* for different values of *w* (the proportion of the total motor variance that arises during motor planning) according to equation 1. The variance is expressed in units of Σ*_mot_*. The location of the minimum (indicated by small circles) depends on the value of *w*. **B** ACF(1) as a function of learning rate *B* for different values of *w* according to equation 2. The learning rate for which the ACF(1) vanishes is equal to the learning rate for which the variance (in **A**) is minimal. **C** Variance plotted as a function of ACF(1) according to Equation 4. This figure shows directly that for each value of *w* the variance is minimal if ACF(1) = 0.

We do not know the value of *w* for dart throwing. We therefore plotted the variance (Figure 2A) and ACF(1) (Figure 2B) as a function of *B* for different values of *w* according to equations 1 and 2. For each value of *w*, there is an optimal learning rate for which the variance is minimal. The location of this optimum depends on *w*; the larger *w*, the larger the optimal value of *B*. Figure 2B shows that for each *w* shown, the ACF(1) is zero for the optimal *B* (see vertical dashed lines). We will now show that is true for every value of *w*.

The simplest way to see this is to determine (1) the *B* for which the variance is minimal from equation 1, and (2) the *B* for which ACF(1) = 0 from equation 2. A straightforward calculation shows that both have the same solution:
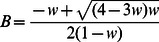
(3)


Alternatively, one could use equation 2 to express *B* as a function of *w* and ACF(1), and then substitute the result into equation 1. After some straightforward but tedious algebra, one finds an expression for the variance as a function of *w* and ACF(1):
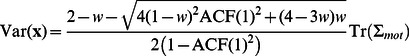
(4)


This relation is plotted for a range of values of *w* in Figure 2C. This figure clearly shows that for each value of *w*, the variance reaches its minimum for ACF(1) = 0. This can also be checked by setting the derivative of equation 4 with respect to ACF(1) to zero.

The result that for every value of *w* the learning rate is optimal if ACF(1) = 0 means that it is not necessary to know the value of *w* to determine whether a participant adopts the optimal learning rate. One only needs to determine the ACF(1). This is the approach we followed here: We measured the ACF(1) of repeated dart throws by experienced and inexperienced dart players to determine whether these groups used the optimal learning rate for trial-by-trial planning corrections.

Note that we cannot be sure that the model for trial-by-trial corrections in reaching [32] also applies to dart throwing. However, it is evident that the principle that the minimum of the variance coincides with the lag-1 autocorrelation being zero holds more generally as any non-zero autocorrelation causes additional variance: a positive ACF(1) leads to random-walk behavior, with its associated variability, whereas a negative ACF(1) corresponds to over-corrections which also induce unnecessarily large variability.

### Participants

We compared two groups of male, right-handed participants. The group of *Experts* consisted of eight dart players from the highest division of local dart leagues (age range: 19–51 years, mean: 34 years, SD: 11 years; dart-experience range: 8–30 years, mean: 16 years, SD: 7 years). The group of *Beginners* consisted of nine undergraduate students who had no dart experience (age range: 20–24 years, mean: 22 years, SD: 1.3 years). All participants were naive to the purpose of the experiment. The experiment was part of a research program that was approved by the ethics committee of the Faculty of Human Movement Sciences of VU University Amsterdam. All participants gave written informed consent before the start of the experiment.

### Apparatus

Each participant performed two tasks, a *Dart task* and a *Reach task*. For the Dart task, we used a sisal dartboard with a diameter of 42.5 cm and 9 black and white concentric rings. In accordance with the rules of the World Darts Federation, the centre of the bullseye was 1.73 m above the floor and the horizontal distance between the dartboard and any part of the participants' shoes was at least 2.37 m. The Experts used their personal darts, whereas standard steel darts were provided to the Beginners. To record the position of the dart on the dartboard, we placed the tip of a pointing device at the required position. This pointing device had six markers, the positions of which were recorded by an Optotrak Certus system (Northern Digital, Waterloo, Ontario). Since the pointing device was pre-calibrated, this allowed us to determine the three-dimensional position of the dart with an accuracy of 0.1 mm.

For the Reach task, participants were seated on a chair and used their right hand to move a stylus on a horizontally-oriented Wacom UD-1825-A drawing tablet (63.5×45.7 cm) that recorded the stylus tip position at 200 Hz with a resolution of 0.1 mm. The participant's arm was not supported against gravity. An LCD projector generated images (1024×768 pixels, 85 Hz) that were displayed on a projection screen above the tablet. Participants looked down onto a mirror that was placed midway between the tablet and the projection screen. They could therefore not see their hand and the stylus, and all images appeared in the plane of the tablet.

### Procedure

Each participant performed the Dart task first, followed by a break of 5 to10 minutes, and then performed the Reach task. In the Dart task, each participant made 180 right-hand throws towards the bullseye (the centre of the dartboard). The dart of the previous throw was left in the dartboard during the next throw so that participants could clearly see the error of the previous throw while planning the next one. After the next throw, the position of the previous throw was recorded, after which that dart was removed from the dartboard. This task took about half an hour to complete.

In the Reach task, participants made 180 reaching movements of the stylus to a visual target. We made this task in several aspects comparable to the Dart task. First, participants did not receive visual feedback about the stylus and their hand during the movements. In this way, participants could not use online visual feedback to control their movement, which is roughly comparable to the ballistic movement of a dart after its release. Second, we gave participants visual feedback about the movement endpoint immediately after each movement, which is similar to seeing where the dart landed on the dartboard.

At the beginning of a trial, a yellow disk (5 mm diameter) appeared against a black background at a fixed location about 35 cm straight in front of the trunk in the mid-sagittal plane. To allow participants to place the stylus quickly and accurately at this starting location and to prevent drift of the felt finger location [35], a red disc (3 mm diameter) was shown at the current stylus location. Once the participant held the stylus still at the starting location, the stylus-location feedback went off and the target appeared. The target was another yellow disk (5 mm diameter) that appeared also in the mid-sagittal plane exactly 10 cm behind the starting location. Participant thus had to move their hand forward, so that participants moved their hand in both tasks in the same direction. The task was to move the stylus as accurately as possible to the target in a single movement. There were no timing constraints. Movements took typically about 400 ms, which is longer than a dart-throwing movement, which takes less than 150 ms [9]. The movement endpoint was determined online as the first location since the start of the movement at which the stylus location was the same in two consecutive frames. From this moment, the movement endpoint was shown for 1 s as a red disc (3 mm diameter) alongside the target. To motivate participants, a score was displayed based on the distance between the endpoint and the centre of the target. The score was inversely proportional to this distance, with a maximum of 100 points that was awarded if the error was less than 1 mm. After the 1 s interval, the target, the score and the endpoint were extinguished, and the starting location and the cursor displaying the current stylus location appeared to start the next trial. Before starting the actual experiment, all participants made ten practice movements (to other targets) to familiarize themselves with the task. This task lasted about 10 minutes.

### Analysis

For both tasks, we analyzed the endpoints of the movements to estimate their lag-1 autocorrelation ACF(1) and variability. For the Dart task, 13 movements (0.42%, 2 of Experts, 11 of Beginners) were excluded from the analysis because their endpoints were not recorded correctly (their recorded positions were more than 5 mm in front of the dartboard). For the Reach task, all endpoints were included in the analysis.

The two-dimensional endpoints will be denoted by (*x* ^(*t*)^, *y* ^(*t*)^), where *t* is the trial number (1, …, 180), and *x* and *y* are the two Cartesian components: For the Dart task, *x* and *y* represent the horizontal and the vertical component, respectively, whereas for the Reach task, *x* and *y* represent the forward and the lateral component. The lag-1 autocorrelation for the *x* component was determined as:
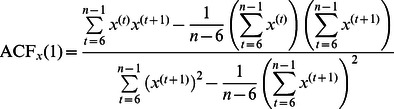
where *n* = 180 is the number of trials in the series. A similar equation was used for the *y* component ACF*_y_*(1). The first 5 movements of a series were not included because correction for a large error in the first movement in the series could occur in these movements [32]; including these movements would lead to an overestimate of the steady-state autocorrelation. The method developed by Marshall [36] was used to deal with missing values. For each participant, we averaged the lag-1 autocorrelations of the two components to obtain the overall lag-1 autocorrelation: ACF(1) = (ACF*_x_*(1)+ACF*_y_*(1))/2.

We used the standard equation for the variance to determine the variance of the *x* and *y* components. The (total) variance was the sum of the variances of these two components.

## Results

### Dart task

**Figure 3:**
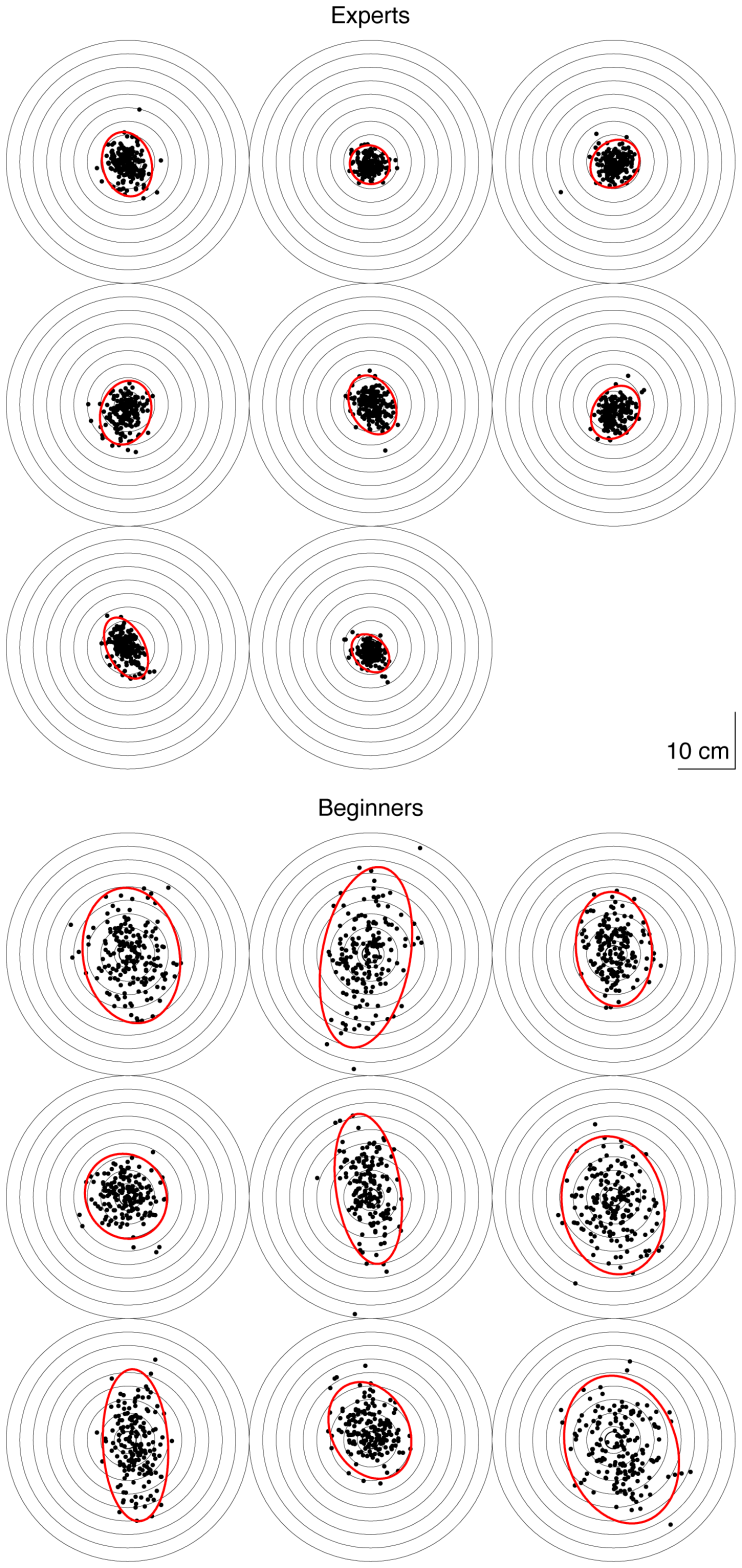
All endpoints of all participants in the Dart task. The circles denote the edges of the concentric rings on the dartboard, dots indicate the positions where the dart landed and red ellipses represent 95% confidence ellipses of the endpoints. All participants hit the dartboard in every trial.

**Figure 4:**
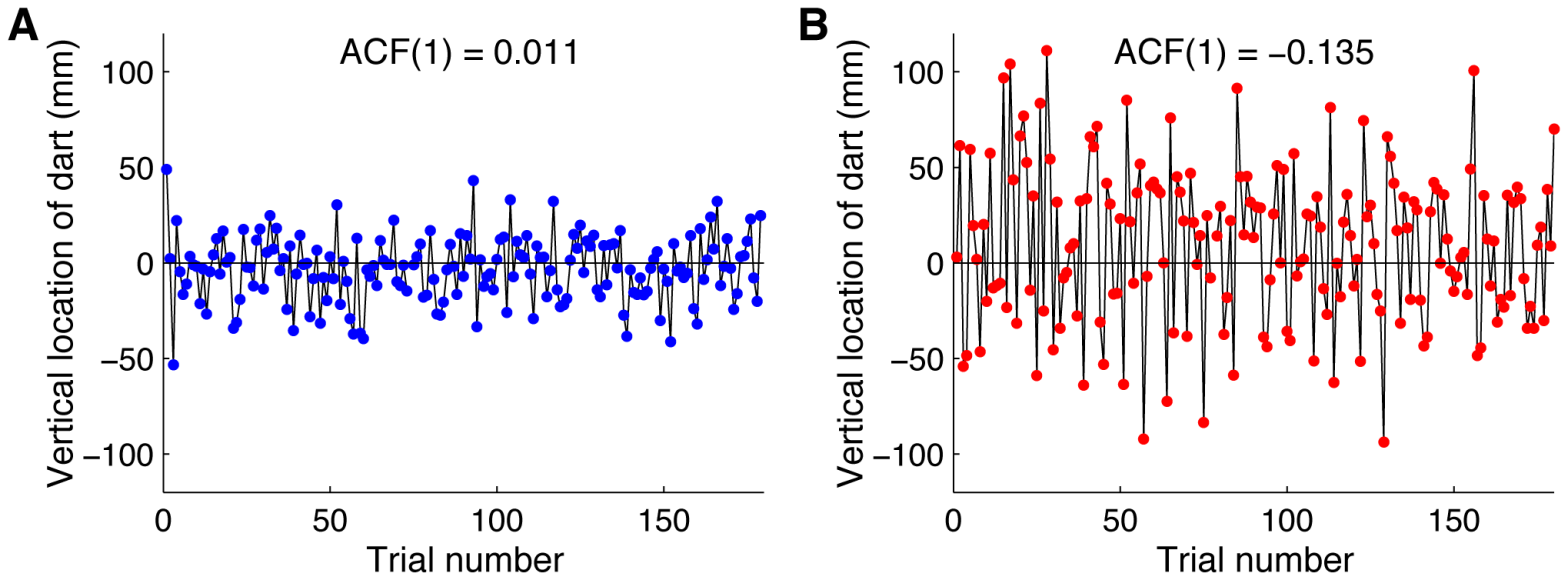
Representative examples of errors as a function of trial number in the Dart task. The vertical component of each endpoint is shown. A value of 0 denotes the bullseye. **A** Data from an Expert. **B** Data from a Beginner.

**Figure 5:**
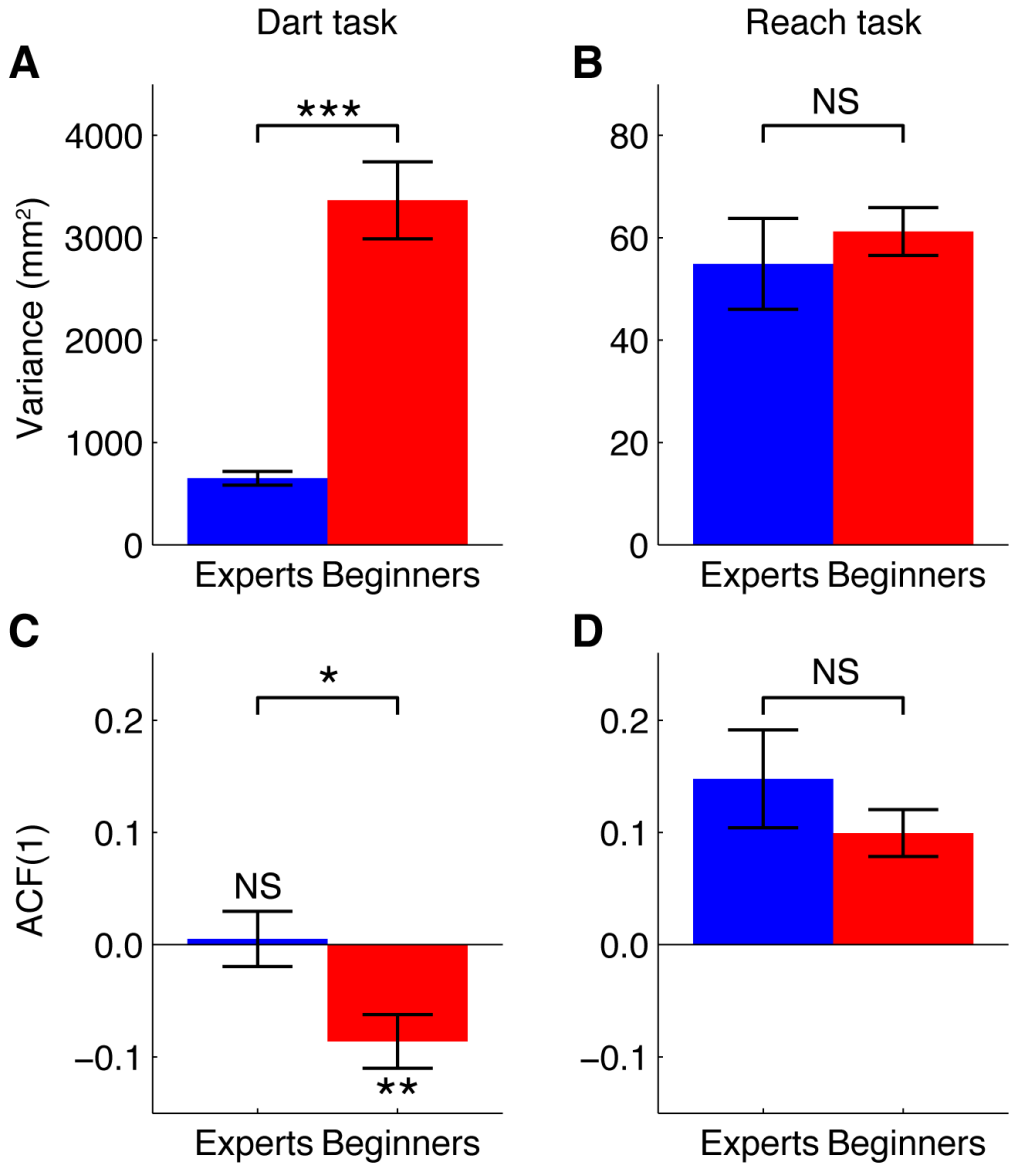
Mean variance and autocorrelation in the two tasks. **A** Variance in the Dart task. **B** Variance in the Reach task. **C** ACF(1) in the Dart task. **D** ACF(1) in the Reach task. In all panels, bars indicate the mean of all participants per group, and error bars denote the between-participant standard error. *: *p*<0.05, **: *p*<0.01, ***: *p*<0.001, NS: *p*>0.05.

Figure 3 shows all the positions where the dart landed on the dartboard, for all participants, and Figure 4 shows representative examples of the vertical positions of the dart as a function of the trial number. Figure 3 shows that the mean endpoint was close to the bullseye for all participants from both groups. This figure also shows that the Experts had a smaller variance than the Beginners (*p* = 3.10^−6^, one-tailed *t* test; see also Figure 5A). This confirms that the Experts were better at this task than the Beginners. For the representative Expert (Figure 4A), there is no clear relation between the positions of consecutive throws, comparable to Figure 1D. This is confirmed by the ACF(1) that was close to zero (0.011). In contrast, for the Beginner (Figure 4B), not only was the variability larger than for the Expert, but there was also a tendency that a positive error was followed by a negative one and vice versa, as in Figure 1F. In agreement with this, the ACF(1) was negative (−0.135). These examples are representative for the two groups (Figure 5C) as the ACF(1) was significantly (*p* = 0.018, two-tailed *t* test) smaller for the Beginners (mean ± SE: −0.086±0.024) than for the Experts (mean ± SE: 0.005±0.025). The ACF(1) was not significantly (*p* = 0.84, two-tailed *t* test) different from zero for the Experts, which suggests that the Experts had near-optimal learning rates for trial-by-trial motor learning. In contrast, the ACF(1) of the Beginners was significantly (*p* = 0.007, two-tailed *t* test) smaller than zero, which indicates that the learning rate used by the Beginners was larger than the optimal learning rate. In other words, the trial-by-trial behavior of the Beginners was such that they over-corrected for observed errors, often jumping over the target (as in Figure 1E, F), giving rise to unnecessarily large endpoint variance. Thus, part of the difference in the variance between the groups (Figure 5A) can be explained by the different groups adopting different learning rates.

**Figure 6:**
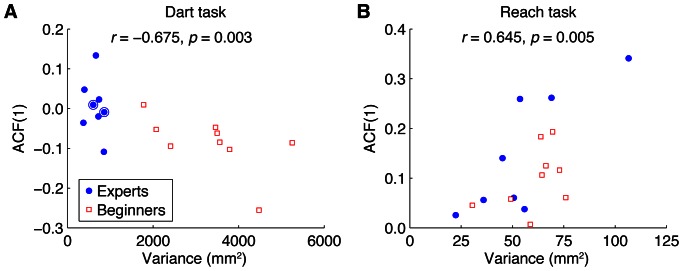
Observed relation between variance and autocorrelation. **A** ACF(1) as a function of variance for the Dart task. Each data point represents a participant. Dots surrounded by a circle indicate the two youngest Experts, who were in the age range of the Beginners. **B** ACF(1) as a function of variance for the Reach task.

Note that the two groups differed not only in dart experience (*p* = 4.10^−6^, one-tailed *t* test) but also in age (*p* = 0.0044, two-tailed *t* test), with the Experts on average being older than the Beginners. One could therefore argue that the difference in autocorrelations might not be related to dart experience but to age. To examine whether this is the case, we looked at the results of the two youngest Experts. These were 19 and 23 years old, well within the age range of the Beginners group. They both had 8 years of darts experience. Figure 6A shows the ACF(1) of each participant as a function of his variance. The data points of the two youngest Experts (indicated by rings) fall within the cluster of data points of the other Experts and are far away from the Beginners' data points. This indicates that it is dart experience, not age, that determines the ACF(1).

We can also look at Figure 6A to examine whether, as suggested above, the variance and ACF(1) are related, such that small variances are found for ACF(1)s near zero and larger variances for ACF(1)s further away from zero. This pattern is visible in the figure, as the largest variances were found for Beginners who had negative ACF(1)s, whereas variances were smaller for Experts, who had near-zero ACF(1)s. The correlation between variance and ACF(1) for the pooled data from both groups in Figure 6A is therefore negative (−0.675) and significantly different from zero (*p* = 0.003). This is however not surprising because we already knew that the two groups had different variances and ACF(1)s. The correlations were not significantly different from zero for the individual groups (Experts: *r* = −0.261, *p* = 0.53; Beginners: *r* = −0.579, *p* = 0.102).

### Reach task

All participants conducted the Reach task to allow us to examine whether the differences found in the Dart task are the result of their differential dart experience or of other differences between the groups. For instance, the Experts could have had an “innate” smaller-than-average learning rate, and therefore have become proficient dart players. If this were the case, this would be evident in a task in which both groups are inexperienced. We chose for reaching with the unseen hand to visual targets because this is, like dart throwing, an approximately ballistic movement that is produced by movement of the dominant arm. Although all participants had a lifelong experience of reaching with their seen hand, they were inexperienced with moving their unseen hand to visual targets.

Figure 5D shows that both groups had a small, positive ACF(1) in the Reach task; the difference between the groups was not significant (*p* = 0.32, two-tailed *t* test). The endpoint variance in this task (Figure 5B) did not differ between the groups either (*p* = 0.52, two-tailed *t* test). These findings suggest that the different ACF(1)s found in the Dart task result from the different amounts of experience with that task and not from other differences between the groups. The observation that the variance in the Reach task did not differ between groups also suggests that the Experts did not have a lower level of “natural” motor variability than the Beginners.

Figure 6B shows the ACF(1) of each participant as a function of his variance in the Reach task. We determined the correlation between these measures to examine whether also in this task a smaller variance corresponds to an ACF(1) nearer to zero. Since all ACF(1)s in this task were positive, the prediction is that the variance and the ACF(1) are correlated positively. This was indeed the case (*r* = 0.645, *p* = 0.005). The correlation was also significant for the Expert group separately (*r* = 0.811, *p* = 0.015), but not for the Beginners group (*r* = 0.452, *p* = 0.222).

### Correlation between tasks

**Figure 7:**
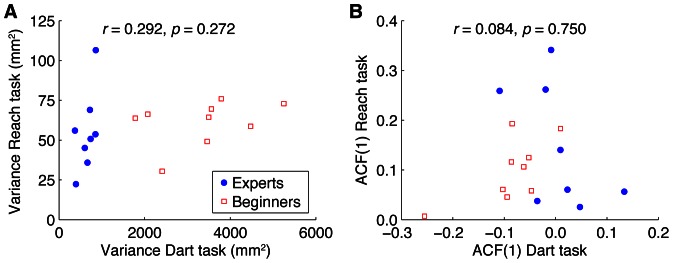
Observed relation between the performance in the two tasks. **A** Variance in the Reach task as a function of variance in the Dart task. Each data point represents a participant. **B** ACF(1) in the Reach task as a function of ACF(1) in the Dart task.

In a final analysis, we examined whether, across participants, the performance variables were correlated between the two tasks. Figure 7A shows the variance in the Reach task as a function of the variance in the Dart task. The correlation between these two variances was not significantly different from zero for the pooled data from both groups (*r* = 0.292, *p* = 0.272), and not for the individual groups either (Experts: *r* = 0.594, *p* = 0.120; Beginners: *r* = 0.415, *p* = 0.306). The ACF(1) in the Reach task is shown as a function of the ACF(1) in the Dart task in Figure 7B. The correlation between these two ACF(1)s was also not significantly different from zero (pooled data from both groups: *r* = 0.084, *p* = 0.750; Experts: *r* = −0.537, *p* = 0.170; Beginners: *r* = 0.474, *p* = 0.235). These results imply that the performance in the two tasks was independent of one another.

## Discussion

Our main finding is that the lag-1 autocorrelation of the endpoints of repeated dart throws was about zero for experienced darters and negative for beginners. The near-zero value for the Experts suggests that these players used the optimal learning rate for translating an observed error into a correction of the motor plan. The Beginners did not do this, as the negative autocorrelation found for this group implies that these players made larger corrections than would have been optimal for minimizing the endpoint variability. Since no differences between the groups were found in the performance of the Reach task, these findings demonstrate that the learning rate of our motor system is not hard-wired and therefore not automatically optimal, but it is optimized through experience. Learning is task specific as the performance in the two tasks was uncorrelated.

One could argue that the near-zero lag-1 autocorrelation of the Experts does not necessarily mean that they made near-optimal trial-by-trial corrections. An alternative explanation could be that their planning did not change from trial to trial at all and was always accurate. The observed variability would then be entirely due to noise in movement execution, which would lead to a zero autocorrelation of movement endpoints, as observed. There are several reasons why this possibility is unlikely. First, this idea has been refuted for reaching movements (Experiment 2 in [32]). That experiment was similar to the Reach task in the present study, but there, participants did not see their actual movement endpoints, as they thought, but they saw endpoints corresponding to half their actual errors. If they did not make trial-by-trial corrections, the autocorrelation would be the same (about zero) as when they saw their actual errors. The observed autocorrelation was however larger when they saw halved errors, suggesting that they did make trial-by-trial corrections, even for very small errors. It seems reasonable to assume that this also applies to throwing by experts as even experts have considerable kinematic variability in their movements [10], [37], [38]. More direct evidence against the idea that expert darters do not change their motor planning from trial to trial is the observation that expert darters make larger errors when feedback about their performance is precluded than when they do get such feedback [38]. This directly demonstrates that they use their seen errors to improve planning of future movements. We conclude that experienced darters do make error-driven trial-by-trial planning corrections, and they do this using a near-optimal learning rate.

The Beginners tended to have negative lag-1 autocorrelations in the Dart task. This was not expected. We expected that Beginners could have autocorrelations further away from zero than the Experts, but we had no expectations about the sign. They could be positive for all Beginners, they could be negative for all, but they could also be highly variable across the population, with some having a positive and others having a negative value. We found that eight out of nine Beginners had a negative autocorrelation, whereas the remaining one had a very small (0.01) positive value. Negative autocorrelations correspond to a learning rate that is larger than the one that minimizes the variance [32]. This leads to over-corrections for observed errors, so that one often jumps over the target (as in Figure 1E,F). The observation that the mean endpoint was close to the target for all Beginners is also consistent with them making substantial trial-by-trial corrections. A possible explanation for the over-corrections is that Beginners attribute too large a part of the error to incorrect motor planning, while underestimating the contribution of noise in motor execution [1], [3], [6]. Ideally, one should correct only for the error arising in motor planning as that will, if left uncorrected, persist in future movements [34], whereas the effects of noise in motor execution are unpredictable and uncorrelated between movements. A negative autocorrelation thus amounts to correcting for (part of) the random effects of execution noise, which is obviously counterproductive. Experienced darters apparently do not do this, but correct, on average, only for the errors arising from inaccurate planning.

Can the between-group difference in the autocorrelation in the Dart task explain the difference in the variance in this task (Figure 5A)? As derived in Materials and Methods, the variance is expected to be lowest if the lag-1 autocorrelation vanishes, and it will increase if the autocorrelation gets smaller or larger than zero (see also Figure 2). The results of both the Dart task (Figure 6A) and the Reach task (Figure 6B) confirm that this relation between autocorrelation and variance exists. However, the difference between the autocorrelations was about 0.1 (Figure 5C), whereas the variance of the Beginners was about 5 times as large as that of the Experts (Figure 5A). Although it is unclear whether the model of van Beers [32] applies to dart throwing, the relation between autocorrelation and variance predicted by this model (Figure 2) suggests that it is highly unlikely that such a small difference in autocorrelation can explain such a large difference in variance. Figure 5B implies that the large between-group difference in the dart variance does not result from differences in the level of “natural” motor variability either, as the variance in the Reach task did not significantly differ between the groups. We therefore conclude that extensive experience with throwing darts not only optimizes the learning rate for trial-by-trial motor learning, but it also reduces the performance variability in other ways. As mentioned in the Introduction, this could be achieved by optimizing the kinematics of the limb movement [2], [8]–[11], [13], [15] and/or by improving the coordination of release parameters [10], [11], [22]–[28].

Our results do not reveal how the optimal learning rate is learned, how many hours of dart throwing are required before one's learning rate begins to change, and how this compares to improvements in other aspects of dart throwing. A longitudinal study is required to address these issues. Note that it is not necessary for a dart player to know the amount of endpoint variability that is caused by motor planning and by movement execution to be able to find the optimal learning rate. One could simply vary the learning rate to see how that affects the performance. If this is tried extensively, the optimal learning rate will be found, as here the average performance will be best. Our results also suggest a strategy for quickly improving a beginner's dart performance. Since a beginner's trial-by-trial corrections tend to be too large, a rapid improvement could be obtained by simply reducing the size of these corrections. Future research could examine whether this strategy leads to an immediate improvement in dart performance.

We conclude by emphasizing the usefulness of the lag-1 autocorrelation as an index of performance in motor-skill learning. Since a lag-1 autocorrelation of zero corresponds to a minimal variance [32], measuring the autocorrelation will directly reveal whether a participant makes optimal trial-by-trial planning corrections, or whether these corrections are suboptimal, leading to unnecessarily large variability. The lag-1 autocorrelation can therefore be used as an index of the optimality of trial-by-trial motor planning. This index can be used in experiments on precision sports, but also in laboratory tasks that study motor-skill learning [39]. In all cases, an autocorrelation of zero indicates optimal performance, whereas a departure from zero implies that performance can be improved. In the latter case, the sign of the autocorrelation indicates whether the size of trial-by-trial corrections should be decreased or increased to improve performance.
